# Retraction: New insights into molecular signalling pathways and current advancements in prostate cancer diagnostics and therapeutics

**DOI:** 10.3389/fonc.2023.1343833

**Published:** 2023-12-07

**Authors:** Frontiers Editorial Office

Following publication, concerns were raised regarding the contribution of the authors of this article. Our investigation, conducted in accordance with Frontiers policies, confirmed a serious breach of our authorship policies and of publication ethics; the article is therefore retracted.

The retraction was approved by the Chief Editors of Oncology and the Chief Executive Editor of Frontiers. The authors do not agree to this retraction.

